# Healthy travel and the socio-economic structure of car commuting in Cambridge, UK: A mixed-methods analysis

**DOI:** 10.1016/j.socscimed.2012.01.042

**Published:** 2012-06

**Authors:** Anna Goodman, Cornelia Guell, Jenna Panter, Natalia R. Jones, David Ogilvie

**Affiliations:** a Faculty of Epidemiology and Population Health, London School of Hygiene and Tropical Medicine, Keppel Street, London WC1E 7HT, UK; b Medical Research Council Epidemiology Unit and UKCRC Centre for Diet and Activity Research (CEDAR), Institute of Public Health, Cambridge, UK; c School of Environmental Sciences, University of East Anglia, UK

**Keywords:** Cars, Travel behaviour, Commuting, Socio-economic factors, Mixed-method, UK

## Abstract

Car use is associated with substantial health and environmental costs but research in deprived populations indicates that car access may also promote psychosocial well-being within car-oriented environments. This mixed-method (quantitative and qualitative) study examined this issue in a more affluent setting, investigating the socio-economic structure of car commuting in Cambridge, UK. Our analyses involved integrating self-reported questionnaire data from 1142 participants in the Commuting and Health in Cambridge study (collected in 2009) and in-depth interviews with 50 participants (collected 2009–2010). Even in Britain's leading ‘cycling city’, cars were a key resource in bridging the gap between individuals' desires and their circumstances. This applied both to long-term life goals such as home ownership and to shorter-term challenges such as illness. Yet car commuting was also subject to constraints, with rush hour traffic pushing drivers to start work earlier and with restrictions on, or charges for, workplace parking pushing drivers towards multimodal journeys (e.g. driving to a ‘park-and-ride’ site then walking). These patterns of car commuting were socio-economically structured in several ways. First, the gradient of housing costs made living near Cambridge more expensive, affecting who could ‘afford’ to cycle and perhaps making cycling the more salient local marker of Bourdieu's class distinction. Nevertheless, cars were generally affordable in this relatively affluent, highly-educated population, reducing the barrier which distance posed to labour-force participation. Finally, having the option of starting work early required flexible hours, a form of job control which in Britain is more common among higher occupational classes. Following a social model of disability, we conclude that socio-economic advantage can make car-oriented environments less disabling via both greater affluence and greater job control, and in ways manifested across the full socio-economic range. This suggests the importance of combining individual-level ‘healthy travel’ interventions with measures aimed at creating travel environments in which all social groups can pursue healthy and satisfying lives.

## Highlights

► This mixed-method study illustrates how cars simultaneously enable and constrain well-being in everyday life. ► Car commuting helped workers in Cambridge (UK) meet life goals (e.g. home ownership) and overcome challenges (e.g. illness). ► Relying on cars also introduced constraints, however, and triggered ‘counter-adaptations’ such as starting work earlier. ► Socio-economic advantage made it easier to achieve sustaining daily routines, via greater affluence and greater job control. ► Thus socio-economic advantage can make car-oriented environments less ‘disabling’, even in a relatively affluent population.

## Introduction

Reducing car use and promoting physically active travel is a public health and environmental priority. Cars generate transport-related air pollution which causes tens of thousands of deaths per year in Europe alone (Krzyzanowski, Kuna-Dibbert, & Schneider, 2005); generate road traffic noise pollution which has been linked with cardiovascular disease and sleep disturbance (WHO, 2007); and generate greenhouse gases which accelerate climate change, set to become a leading public health issue (McMichael, Woodruff, & Hales, 2006). Cars are also major contributors to road traffic injuries, predicted to become the third largest contributor to the global burden of disease by 2020 (WHO/World Bank, 2004). These costs are largely borne by individuals other than those travelling within the car, disproportionately affecting socio-economically disadvantaged individuals and communities (Hasselberg, Vaez, & Laflamme, 2005; Mitchell & Dorling, 2003; Woodcock & Aldred, 2008). Car travel additionally imposes upon car users the direct health cost of enforced sedentary time and the opportunity health cost of forgoing more physically active travel modes (e.g. walking or cycling) (Frank, Saelens, Powell, & Chapman, 2007; Wen, Orr, Millett, & Rissel, 2006). Active travel has therefore received increasing policy interest as one means of integrating physical activity into everyday life (WHO, 2002).

Yet there is a paradox in the relationship between cars and health. On the one hand, within groups of (otherwise broadly similar) commuters, those who commute by car are more likely to be overweight or obese (Lindstrom, 2008; Wen et al., 2006), while those who walk or cycle are more likely to meet recommended levels of physical activity (Lindstrom, 2008; Wen et al., 2006), enjoy better cardiovascular health (Hamer & Chida, 2008) and in some studies have lower mortality (Andersen, Schnohr, Schroll, & Hein, 2000; Matthews, Jurj, Shu, Li, Yang, Li et al., 2007). Across the whole British population, however, social epidemiological surveys consistently find that car ownership is associated with better health, including lower rates of mortality (Smith & Harding, 1997), chronic diseases (Ferrie, Shipley, Breeze, & Davey Smith, 2006), long-term illness (Macintyre, Hiscock, Kearns, & Ellaway, 2001) disability (Ebrahim, Papacosta, Wannamethee, & Adamson, 2004) and mental health problems (Macintyre et al., 2001). This is partly explained by the social gradient in car ownership: for example, in 2009 52% of British households in the bottom fifth of the income distribution had no car, compared to only 10% in the highest fifth (Donabie, 2011). The social gradient in car ownership appears not to be the full explanation, however, as the association persists after adjusting for factors such as employment status, home ownership or occupational social class (reviewed in Ellaway, Macintyre, Hiscock, & Kearns, 2003).

The independent effect of car ownership may reflect the physical and psychosocial health costs of being car-less in an environment predicated upon the assumption of universal car access. In the past 50 years, cities around the world have become less compact (more ‘sprawled’), and within Europe levels of urban sprawl are particularly high in Northern and Western Europe (although still lower than in Australia and the US) (EEA, 2006). Such urban sprawl is strongly associated with greater energy consumption from private vehicles and with reduced walking or cycling to work (Newman & Kenworthy, 1999). Freund and Martin (2001, 2004) argue that such highly-sprawled car-oriented environments can ‘disable’ those without cars (see also Aldred & Woodcock, 2008), following a social model of disability as a “social experience [arising] from the specific ways in which society organizes its fundamental activities (i.e. work, transport...)” (Gleeson, 1999: 25). For example, a qualitative study in the Midlands highlighted the fatigue and stress faced by car-less, low-income mothers in daily life, such as when walking to distant shops with fractious children in tow (Bostock, 2001). As Bostock describes, these mothers were forced “to substitute their time and labour for goods and services [like cars] that can ease the workload of life on a low income. In effect, mothers used their bodies as a means to bridge the gap between responsibilities and resources” (p.16). A further source of stress was dependency upon favours from car-owning friends and relatives, undermining their autonomy and leading some to forgo social visits or healthcare appointments. Reduced autonomy also emerged as a theme in qualitative and quantitative studies in relatively deprived parts of Scotland, together with reduced prestige and increased vulnerability to undesirable people or events (Ellaway et al., 2003; Hiscock, Macintyre, Kearns, & Ellaway, 2002). From these findings, the authors argue that car access enhances ontological security which, drawing on the work of Laing (1960) and Giddens (1991), they define as “a long term tendency to believe that things are reliable and secure as opposed to threatening” (Ellaway et al., 2003: 221).

Car-oriented environments may thus increase health inequalities both because poorer groups disproportionately bear many of cars' health costs and also because poorer groups more often face the disabling effects of being car-less in a car-oriented environment. This may explain why previous studies have focussed upon relatively deprived populations when examining the socio-economic structure of car use and its implications for health. Nevertheless we believe there are several important reasons for also studying more affluent groups. First, socio-economically advantaged groups contribute most to the health and environmental costs of cars (Brand & Boardman, 2008; Edwards, Roberts, Green, & Lutchmun, 2006) and are arguably therefore particularly important target populations for interventions. Secondly, socio-economic groups may differ markedly in the practical and symbolic benefits they ascribe to travel modes (Steinbach, Green, Datta, & Edwards, 2011). Finally, many leading causes of health inequalities do not operate only upon the poorest groups, but rather generate inequalities across the full socio-economic range (WHO, 2008). As we argue below, this may also apply to individuals' ability to pursue health and well-being within car-oriented environments. The aim of this study was therefore to examine the predictors of car commuting in a comparatively affluent population, with a particular focus upon the role of socio-economic position. Unusually in this field, we sought to address this aim by integrating qualitative and quantitative data, seeking thereby to strengthen our inferences and expand the scope of our research (Stewart, Makwarimba, Barnfather, Letourneau, & Neufeld, 2008).

## Methods

### Setting

Our study setting is the city of Cambridge, UK (population 109,000) and the surrounding towns, villages and rural areas (population 494,000 in study catchment area: Census, 2001). The comparative affluence of this setting is demonstrated by examining small-area income deprivation (Department for Communities and Local Government, 2011): 41% of small census area units (lower super output areas) in our study catchment area were in the least deprived fifth in England vs. only 1% in the most deprived fifth. This privileged socio-economic profile of Cambridge residents is matched by an overrepresentation in the labour market of highly-skilled professions, for example in higher education or the science and technology sector.

Cambridge also stands out as Britain's leading ‘cycling city’, reflecting its compact nature, flat topography, congested city centre, high student population and active cycling lobby. In the 2001 census, 28% of Cambridge city residents commuted by bicycle vs. 5% in the surrounding areas and 3% in England (Census, 2001). Even within the city, however, cycle commuting was less prevalent than car commuting (43%), and in the surrounding areas car commuting was more prevalent than the national average (77% vs. 67% in England). Thus while Cambridge city is less car-oriented than other parts of Britain in relative terms, it remains car-oriented in absolute terms, while the surrounding areas are car-oriented even in relative terms. For those who do commute by car, Cambridge has limited street parking but has five ‘park-and-ride’ sites plus one ‘park-and-cycle’ site for University employees. In Cambridge, these sites provide free daytime car parking on the edge of the city and allow people to continue their journeys on foot, by bicycle or by catching one of the dedicated bus services (for which there is a charge).

### Participants

*Commuting and Health in Cambridge* is an ongoing cohort study described elsewhere (Ogilvie et al., 2010). Participants were aged 16 or above, lived within 30 km of central Cambridge and commuted to pre-specified Cambridge workplaces. Recruitment took place through workplaces but (for data protection and to assure participants of the study's independence) did not use employer-based sampling frames such as staff databases. Instead employees were invited to opt in through strategies such as recruitment stands, advertisements and emails. All participants provided written informed consent and the Hertfordshire Research Ethics Committee granted ethical approval.

Of 1164 participants who completed questionnaires in the first phase of the study in 2009, we excluded 22 who recorded no past-week commute journeys. Our quantitative study population therefore comprised 1142 individuals (17–71 years, 782 females). Of these, 50 (21–69 years, 29 females) subsequently completed in-depth qualitative interviews in 2009–2010.

### Mixed-method approach

**Fig. 1:**
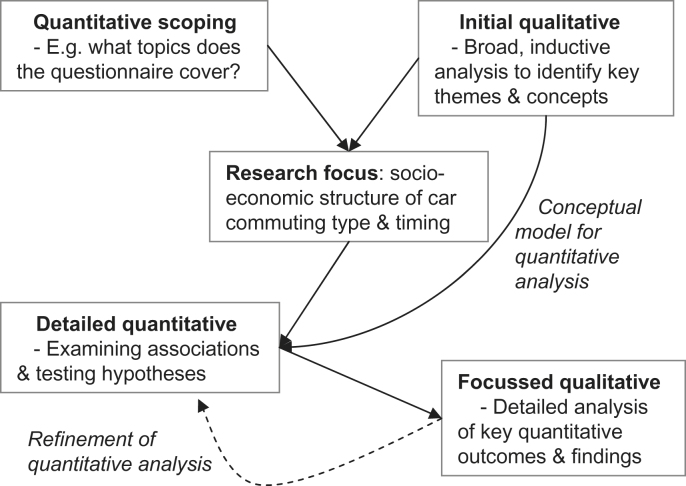
Process of mixed-methods integration.

We pursued our qualitative and quantitative research components in parallel (a concurrent mixed-method design), aiming to integrate these research strands when designing our methods, analysing our data, and interpreting our results (Fig. 1). In the methods stage, this integration involved using initial qualitative findings to design our conceptual model for statistical analysis and to suggest additional lines of enquiry. At the analysis stage this involved pursuing key themes between datasets, an approach described by Moran-Ellis et al. (2006) as ‘following a thread’. We thereby attempted to achieve a ‘genuinely integrated’ mixed-method study, allowing our quantitative and qualitative findings “to talk to each other and...[construct] a negotiated account of what they mean together” (Bryman, 2007: 21).

### Qualitative sampling and analysis

Qualitative participants were drawn from among those who completed survey questionnaires in 2009, and were selected purposively to generate a diverse sample in terms of age, gender, place of residence and commute mode. Semi-structured interviews were conducted at participants' homes, workplaces or other convenient locations, and lasted between 20 and 60 min. The interviews were guided by flexible topic guides covering the participant's typical commute to work, any variations upon their normal routine, and the factors shaping these commuting behaviours and decisions. Some participants also completed a follow-up interview several months later, resulting in 68 interviews from 50 participants. Interviews were taped and transcribed verbatim, and were contextualised through interviewer field notes. NVivo 8 was used to facilitate data management and coding.

Qualitative analysis was led by AG with peer-checking by the researchers who conducted the interviews (NJ in 2009, CG in 2010). The initial ‘broad’ analysis phase (see Fig. 1) involved micro-level open coding, the identification of emerging themes and conceptual categories, and an iterative approach which refined these categories through discussion and ongoing analysis. The second analysis phase involved a more focussed content analysis which designed and applied coding schemes based around the key quantitative outcomes and findings (e.g. ‘regular car commuting’ or ‘commute timing’). In presenting quotes, names have been changed and identifying details removed.

### Quantitative outcomes and explanatory variables

Participants retrospectively reported their work start time, end time and commute modes on each of the past seven days. Participants also reported how often in the past four weeks they had travelled to work by car/motorbike, bicycle, walking, and public transport (response options ‘always’, ‘usually’, ‘occasionally’ or ‘rarely or never’). Responses to the car/motorbike item were assumed to refer to cars unless the participant reported more past-week commuting trips by motorbike than car (*N* = 10). Finally participants reported whether they ever commuted by car, including as a passenger. We recoded this variable to ‘yes’ for 35 individuals who answered ‘no’ but reported past-week car commuting.

Our primary outcome variable was regular car commuting, defined as usually or always (vs. occasionally, rarely or never) commuting by car in the past four weeks. As a secondary outcome, we subdivided regular car commuters according to whether over 50% of past-week car trips were ‘unimodal’ (involving only one travel mode, i.e. driving all the way) vs. ‘multimodal’ (involving cars plus another mode or modes, e.g. park-and-ride). We also divided non-regular car commuters into ‘occasional’ vs. ‘never’ car commuters, according to whether they reported ever commuting by car.

Table 1 presents the potential predictors of car commuting which we examined. All variables were self-reported, except for small-area income deprivation which we assigned using home postcodes and the 2010 Indices of Multiple Deprivation (Department for Communities and Local Government, 2011). We combined 101 participants reporting ‘other’ educational qualifications with the ‘degree’ category (*N* = 788) because these ‘other’ qualifications were postgraduate qualifications in all 31 cases where a comment was provided. Our findings were unchanged in sensitivity analyses treating ‘other’ qualifications as a separate group.

### Conceptual model and statistical analysis

We used our initial qualitative analyses to develop a conceptual model for our statistical analyses. This model hypothesised that demographic, health and socio-economic characteristics would predict how far participants lived from their workplace, and that the effects of these characteristics upon car commuting might therefore be partly mediated by commute distance. It also seemed plausible that commute distance would in turn be partly mediated by car access, for example because participants responded to greater distance by buying an additional car. We further hypothesised that demographic, health and socio-economic differences might be directly mediated by access to cars or workplace parking, the latter because some workplaces only provided parking for senior-level employees or employees with young children.

We examined these hypotheses by fitting a hierarchical series of regression models, using Poisson regression with robust standard errors (Zou, 2004). We used multiple imputation (five imputation models) to impute missing data (≤1% for all variables) under an assumption of missing at random.

Finally, we examined the association between commute mode and commute timing in the past-week commuting diary. For this we used days as our units of analysis, calculating robust standard errors to allow for clustering within participants. All analyses were conducted in Stata 11.1.

## Results

**Fig. 2:**
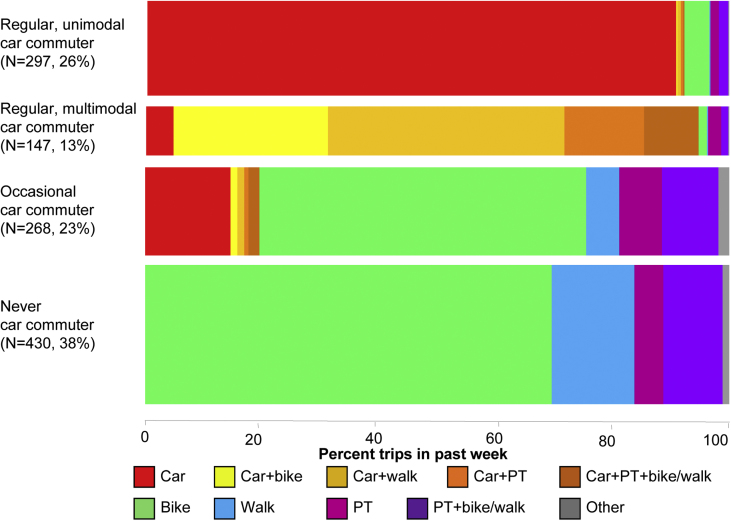
Car commuting type in relation to past-week commute trips. PT = public transport. Data tabulated in the Electronic appendix available with the online version of the paper.

As shown in Fig. 2, there was a strong association between car commuting type and past-week commute modes (*p* < 0.001). There were consequently also strong associations between car commuting type and active travel: only 5% of journeys by the 297 regular unimodal car commuters involved any walking or cycling, vs. 80% in the 147 regular multimodal car commuters, 76% in the 268 occasional car commuters and 94% in the 430 who never commuted by car.

### Predictors of regular car commuting

Table 1 presents the individual, household and work-related characteristics of our participants, and Table 2 examines associations with regular (vs. non-regular) car commuting. Regular car commuting was more common in women and participants aged over 30, but this age effect disappeared after adjusting for socio-economic position (SEP), particularly housing tenure. The minimally-adjusted effects of long-term limiting illness and difficulty walking were likewise attenuated and became marginally significant (*p* = 0.04) after adjusting for SEP. There was no evidence that having children predicted regular car commuting.

The three SEP markers showed strong minimally-adjusted effects in conflicting directions: regular car commuting was negatively associated with education, positively associated with home ownership and showed an inverted U-shape association with income deprivation. These effects changed little after adjusting for all demographic, health and socio-economic characteristics (all *p* < 0.001: multivariable model 1 in Table 2) but were attenuated to the null after additionally adjusting for commute distance (all *p* ≥ 0.10: model 2). Commute distance had a very strong effect: 6% of those living within 5 km of work regularly commuted by car vs. 72% of those living over 10 km away. This effect diminished only slightly after adjusting for having a driving licence, household cars and workplace parking, which all also had independent effects (model 3).

These results therefore suggested that the effect of age upon regular car commuting was mediated by SEP (particularly housing tenure), and that SEP effects were in turn mediated by commute distance. Further analysis suggested that this reflected aspects of the local geography, including distinctive Cambridge migration patterns and lifecourses. Comparisons with Census (2001) data indicated that our study population was not representative of the general residential population, containing a higher proportion of women and those aged over 30, those with degree-level education and those living in privately rented accommodation. Nevertheless our sample and the Census data showed a similar relative spatial patterning of these socio-demographic characteristics, with Cambridge city residents being younger, better educated and more often privately renting than those in the surrounding areas (see Electronic appendix available with the online version of the paper). This constellation of characteristics plausibly reflects an overrepresentation of highly-skilled employees renting and perhaps sharing houses in Cambridge city. In qualitative interviews, those who were older or wished to settle more permanently in Cambridge often described moving further out to afford to buy a house or improve their living standards. This came out particularly clearly in the lifecourse narratives of longer-term Cambridge residents:

“We've lived in Cambridge for a long time, we met at university […] and lived in Cambridge in shared houses for a while.

[…T]hen we found out that they were selling off all the ex-Ministry of Defence properties in [village 9 km from Cambridge...and] because it was the only way we could afford to buy a house at the time, we jumped on the chance.” (Kate, age 35, multimodal car commuter in quantitative survey but subsequently switched to cycling)

“We wanted a larger village with a community spirit and we were looking a ten mile radius, couldn't find much, housing situation being what it was [...so we] widened the scope to a fifteen mile radius. Nearly didn't go, well no actually we found the right house but it had never been our intention to actually have to travel on the A14 [a main road linking Cambridge to nearby towns] each day.” (Susan, age 58, unimodal car commuter)

“Because everything's so expensive here, people who I was mixing with were having to move further out to get accommodation therefore you needed a car. To be honest, not having a car in Cambridge and being able to survive, it's indicative of quite a wealthy lifestyle because house prices are so expensive. It is an odd factor about Cambridge that you'll find, if you can afford to, you're only cycling three miles you've probably got quite an expensive house [...or] two or three of you paying the rent on a property.” (Matt, age 44, multimodal car commuter)

Both the quantitative and qualitative data therefore suggested that individuals' life stage and socio-economic circumstances influenced how far they lived from Cambridge, which in turn affected travel behaviours. Moreover place of residence seemed *fully* to explain this relationship in the quantitative analyses, with no independent SEP effects after adjusting for commute distance (Table 2). This lack of direct SEP effects may reflect the affordability of cars among our participants. For example, only 32/481 (7%) of participants living 10 km from their work had no car in their household (*N* = 16) and/or no driving licence (*N* = 27). Two of these 32 participants completed a qualitative interview and explained that they used the bus to avoid ‘bothering’ to get a driving licence. Likewise, although many qualitative participants discussed the costs of different commuting options, none said they would strongly prefer car commuting but could not afford this.

In contrast to Britain as a whole, therefore, not owning or commuting by car was not generally a marker of deprivation in this population. On the contrary, as suggested by Matt above, it may more often have reflected being rich or fortunate enough to live near enough to work to cycle – a finding consistent with our previous quantitative demonstration that highly-educated participants living close to work were more likely to cycle (Panter, Griffin, Jones, Mackett, & Ogilvie, 2011). Nevertheless the general affordability of cars meant that even those living further away could travel to work without placing undue strain upon their time, bodies, or loved ones:

“I did try the bus for a while when one of my cars, I think, broke […but] it was taking an hour of my time every way so it was a waste of, well an hour and a half really.” (Catherine, age 46, multimodal car commuter)

“I used to try to cycle to work but I moved house, so I moved further north, so the distance from work was slightly greater […] But it wasn't just that, it's the roads are treacherous around there basically, and I wouldn't feel safe cycling.” (David, age 35, unimodal car commuter)

“Before I had my daughter, I used to drive in and then get the park-and-ride [...but that] would mean getting her up earlier, more pressure in the morning […]. It's probably more an issue with coming home because if the bus doesn't arrive, you've got a very tired crabby toddler who doesn't want to stand, who doesn't want to wait, maybe waiting an hour for a bus.” (Erica, age 37, unimodal car commuter)

Car commuting thus enabled many to achieve life goals such as home ownership (a strong social norm in Britain) while maintaining their careers, thereby bridging the gap between individuals' desires and their circumstances. In this sense car commuting was an adaptation, increasing individuals' abilities to achieve well-being within a particular, car-oriented environment. Using car commuting to transcend distance did, however, leave many participants highly reliant upon cars, as illustrated by the disruptive effects of interruptions to their routine:

“[If my normal car share isn't possible then] I can't come in on the bus, it just wouldn't get me here on time, I have patients. I can't, I can't be late so I would have to organise something like getting up at 5am to take my husband to work to bring the car back and then bring the car, or I will phone the other friend and say “please can I have a lift today?”” (Vivienne, age 57, unimodal car commuter)

### Predictors of multimodal car commuting

As Table 3 shows, only workplace parking strongly predicted unimodal (vs. multimodal) car commuting: 90% of regular car commuters with free workplace parking reported unimodal commuting vs. 64% who had to pay and 19% without workplace parking (these 19% may have parked on adjacent streets or been dropped off by others). The cost and/or reliability of workplace parking were likewise prominent in the qualitative data, and even those who identified other benefits of their multimodal commuting (e.g. enjoying the walk) sometimes indicated elsewhere in their interview that unimodal car commuting would be the ‘obvious’ choice if suitable workplace parking were available. One multimodal commuter did, however, explain that he had not applied for free onsite parking precisely *because* he wished to retain the health benefits of cycling. Thus although workplace parking generally appeared a key determinant of unimodal car commuting, the direction of causality may sometimes have been reversed.

### Predictors of occasional car commuting

Among non-regular car commuters, the independent predictors of occasional (vs. never) car commuting were female gender, longer commute distance, more household cars and access to workplace parking, particularly free parking (Table 3). Home ownership and difficulty walking were also strong predictors in minimally-adjusted analyses, but became non-significant after adjusting for commute distance. Occasional car commuting was therefore broadly similar to regular car commuting in its quantitative correlates. Qualitative analysis indicated further conceptual similarities, with occasional car commuting again representing an adaptive response that could bridge the gap between circumstances and desires. Specifically, many participants valued occasional car commuting as a way to fulfil working, caring or personal commitments despite everyday challenges such as illness, icy weather or transporting heavy equipment:

“Because my wife's not ever so well at the moment [...] I'm getting up early with the dog and then by the time I've sorted all that out it's then probably too tiring to bike to work.” (James, age 42, occasional car commuter)

“Especially with the little one on the back, but even without him I wouldn't cycle in the ice...I tried a number of things. I tried getting the bus and I tried driving halfway and walking halfway but, I have to be honest that in the end I just drove, cos it was just too much hassle.” (Becca, age 34, occasional car commuter)

Moreover, and again reflecting the comparative affluence of this sample, some of those without cars occasionally used taxis for the same reasons. Thus just as regular car commuting enabled participants to meet long-term goals such as home ownership, occasional car (or taxi) commuting allowed participants to rise to short-term challenges without wasting time or exposing themselves to risk or stress. Similarly in the medium-term, car commuting might temporarily allow participants to adapt to challenges associated with particular phases of the lifecourse:

“Well in that case, it was really a necessity [to drive], because our son hadn't started school, our childminder was too far away to cycle […] and there was no bus connection from us to the childminder. So for that period of time when my son was six months old till now, school starting, it was really necessary to take the car.” (Paula, age 40, occasional car commuter)

### Commute mode and commute timing

One theme emerging from our initial qualitative analysis was the effort most commuters made to avoid sharing physical and social space, creating an effortful and artificial a-sociality which we termed a ‘choreography of avoidance’. In space, this choreography affected all travel modes and seemed primarily to be about creating a safe and/or pleasant journey: for example, choosing cycle routes away from cars or pedestrians; avoiding groups of school children on public transport; or preferring cars to public transport in order to have one's own space. By contrast, the choreography of avoidance in *time* primarily affected car and public transport commuters, who frequently described leaving early in order to avoid the delay and unpredictability of travelling in rush hour or, less frequently, in order to find a parking space. These considerations generally did not affect walkers or cyclists, who typically described their journey duration as far more predictable and far less vulnerable to external circumstances.

**Fig. 3:**
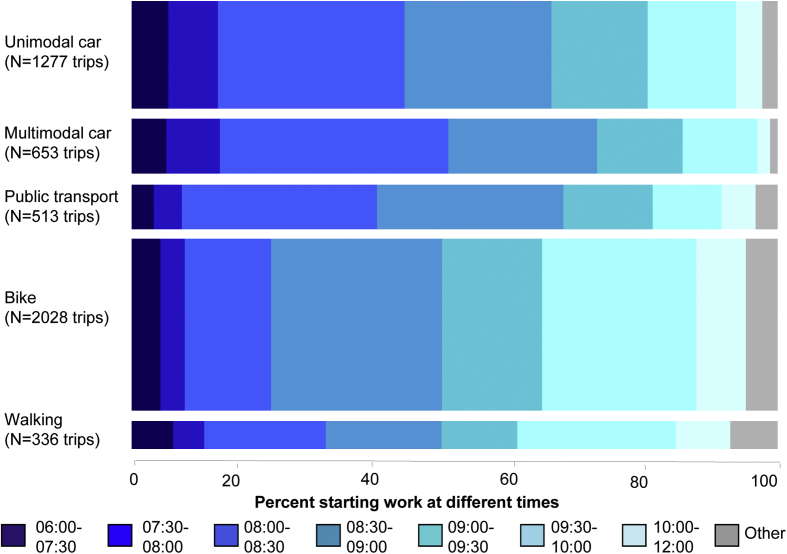
Association between commute mode and time of starting work. PT = public transport. Data tabulated in the Electronic appendix available with the online version of the paper.

We pursued this ‘thread’ into the quantitative dataset using the past-week commuting diaries. There was no evidence that car commute type was associated with the time participants spent at work (*p* = 0.31) or their overall number of commute trips (*p* = 0.46). There was, however, strong evidence that car and public transport commuters started work earlier (e.g. around 40% arriving by 8:30am vs. 20% of walkers or cyclists: see Fig. 3) an association which persisted after adjusting for the demographic, socio-economic and workplace characteristics presented above (*p* < 0.001 for heterogeneity).

Thus despite enabling short- and long-term adaptations to a car-oriented environment, car commuting also pushed people towards the ‘counter-adaptation’ of starting work early to reduce their journey time and/or to make it more predictable. Further qualitative analysis highlighted the key role played by flexible working hours in pursuing this counter-adaptation, and also in combining commuting with other commitments (e.g. caring for a parent) or overcoming disruptions to one's routine (contrast with Vivienne's quote above). Those without flexible hours therefore sometimes regretted or resented this:

“Participant: They don't have flexitime, I've always worked places where, you know, if you come in and you work from 8 till 6 then you can claim the extra hours and take them off and here, it's like *(gasps)* revolutionary and you just think, oh, you know.

Interviewer: Yeah, and that would make it easier to travel...

Participant: I think it would make it a lot easier. Yes, it would make it a lot easier but they're not into that. And anyway they don't regard assistant staff as like normal people.” (Fran, age 62, unimodal car commuter)

In linking her lack of flexitime to the fact that she was only an assistant staff member, Fran highlighted that the option of early car commuting may require a degree of job control not available to all employees. Several other participants similarly linked their flexible working hours to the professional nature of their jobs, and national British surveys (Nisar, 2006) support Fran's suggestion that access to flexitime is particularly common among senior-level and professional employees.

## Discussion

This mixed-method investigation of 1142 adult commuters in Cambridge, UK, has argued that car commuting is an adaptation that facilitates well-being by allowing individuals to achieve long-term goals such as home ownership, and to negotiate short- and medium-term challenges such as illness or organising childcare. Car commuting is itself subject to constraints, however, and car commuters pursue counter-adaptations such as starting work early. Both these adaptations and counter-adaptations are socio-economically structured, reflecting the role of house price gradients upon commute distances; the general affordability of cars (or occasional taxis) in this relatively affluent population; and perhaps the flexible working hours common in the professional occupations held by many participants.

Before discussing these findings further, it is worth considering the strengths and limitations of this research. One key strength is our complementary use of qualitative and quantitative data: for example, our qualitative data allowed us to situate car commuting within the lifecourse, while our quantitative analyses brought out the role of structural factors such as SEP. Our use of multiple SEP markers is a further strength, and the conflicting directions of their minimally-adjusted effects highlights the danger of relying upon single SEP markers in social science research. Nevertheless this study has important limitations. Its use of selected workplaces and opt-in recruitment means our quantitative sample cannot be assumed to be representative of Cambridge commuters. Yet although the socio-demographic profile of our sample differed from that of the general resident population in absolute terms, the relative spatial patterning was broadly similar. This suggests that the associations we have presented may be less affected by selection bias than the absolute prevalence values. Our reliance on cross-sectional quantitative data prevented us from examining the direction of causality between car commuting and its correlates. For most of the correlates presented in this paper reverse causality seems impossible (e.g. age), implausible (e.g. SEP, number of children) or else was suggested by the qualitative data as playing only a minor role (e.g. commute distance). Difficulties of causal attribution did, however, lead us to omit other potential correlates such as weight status or self-reported mental health, a limitation which we intend to redress once longitudinal quantitative data become available. We also lacked quantitative data on factors such as life satisfaction or autonomy which could have complemented our qualitative analysis of how car commuting may promote psychological well-being. Finally, the importance of local circumstances in understanding mobility cultures (Steinbach et al., 2011) means our analysis should be seen as a case study of one particular city. Nevertheless the combination of high inner-city house prices and sufficient affluence to afford car commuting has been identified as a major contributor to urban sprawl (and concomitant car dependency) in numerous European cities (EEA, 2006). This suggests the potential generalisability of some of our socio-economic findings to other settings with similar geographies.

We also believe the broader conceptual principles may provide useful insight in a wider range of settings. Recent work in the anthropology of well-being argues that all individuals and families desire to “construct a social ecology that balances what they want [...with] what is possible in their circumstances. This involves sustaining a daily routine” (Weisner, 2009: 228). This study illustrates how car commuting can facilitate this, while also allowing individuals to meet challenges when their routine is interrupted. This supports and extends previous research which argues that car access provides psychological benefits by enhancing ‘ontological security’ in less affluent populations (Ellaway et al., 2003; Hiscock et al., 2002), aspects of which include believing that one inhabits a stable world and can overcome life's obstacles. Enabling daily routines which are both sustaining and ‘shock-resistant’ therefore appear two key routes whereby car access can promote well-being in a car-oriented environment. The fact that this applies even in Britain's leading cycling city underlines how car-oriented Britain is and (together with this replication in a more affluent population) suggests these effects may be widespread.

Nevertheless, using car commuting to transcend distance left many participants highly reliant on their cars, sometimes reluctantly abandoning the health benefits and even greater predictability of their former cycling. Car commuting also pushed many towards the counter-adaptation of starting work earlier. This highlights how car-oriented transport systems can affect the social organization of space and time, the health consequences of which previous analyses have largely addressed with reference to space (Freund & Martin, 2001, 2004; Woodcock & Aldred, 2008). For example Freund and Martin (2004: 277) argue “Increasingly, exercise is confined to workouts that aim to maximise the productivity of fitness activity. The time available for playful uses of the body...has shrunk. [...] This time squeeze is aggravated by car-hegemonic organisations of movement space, particularly their tendency to disperse and to sever activity sites, including fitness sites (public parks, footpaths...)”. The earlier start times of car commuters indicates how car-hegemonic transport systems can also affect movement *time*, arguably aggravating the time squeeze still further.

From a public health perspective, a further counter-adaptation of particular interest lies in the strong effects of workplace parking upon all forms of car commuting and especially upon regular car commuters' choice of unimodal vs. multimodal commuting. This impact of workplace parking upon car commuting is consistent with previous studies (reviewed in Wen, Kite, & Rissel, 2010), and extends these by highlighting the implications for active travel. If these active travel differences translate into differences in total physical activity, it suggests the potential health benefits of restricting or charging for workplace parking, whilst simultaneously providing facilities such as park-and-ride sites.

Many of the above adaptations and counter-adaptations were shaped by socio-economic structure. In contrast to less affluent populations (Ellaway et al., 2003; Hiscock et al., 2002), car ownership did not seem to be a source of social status in this setting. Indeed, if anything cycling rather than car ownership appeared a more salient local marker of Bourdieu's (1978) class distinction, echoing recent findings from London (Steinbach et al., 2011). This highlights a potential class dimension to individuals' ability to practice ‘cyclising citizenship’ in Cambridge (Aldred, 2010), to adopt active commuting and/or to choose not to use a car despite living in a car-oriented environment. Yet although many participants could not afford to live in Cambridge city, cars were generally affordable and flexible working hours were relatively common. These factors reduced the barriers to labour-force participation, allowing participants to combine personal, caring and work roles without placing undue demands upon themselves or their families. This liberating role of socio-economic advantage is underscored through comparisons with research in deprived populations. For example, while Bostock (2001) describes low-income mothers struggling to walk with their young children because they could not afford the bus, parents in our sample could not only afford buses but frequently chose the still greater convenience of cars. Cars thus represented an additional resource for our participants in meeting their everyday responsibilities, supplementing and relieving the pressure on their time, bodies and social networks.

Drawing on a social model of disability (Freund & Martin, 2004), we therefore conclude that socio-economic advantage makes car-oriented environments less disabling in ways manifested through both greater affluence and greater control. These socio-economic effects are particularly striking when comparing this relatively affluent population to more deprived groups elsewhere. These effects operate even within this population, however, and at the most affluent extreme include the ability to negotiate a car-oriented environment *without* relying on cars. This study of car commuting therefore provides an example of how socio-economic inequalities in health and well-being may operate across the full socio-economic range and not simply upon society's poorest groups (WHO, 2008). It also suggests the importance of combining individual-level ‘healthy travel’ interventions (e.g. restricting workplace parking) with broader measures aimed at making environments less car-oriented. In this way it may be possible to achieve a population-level shift away from cars while at the same time protecting and extending the ability of all social groups to pursue healthy and satisfying lives.

## Competing interests

None.

## Figures and Tables

**Table 1:** Individual, household and work-related characteristics of study participants (*N* = 1142).

			*N* (%)
Demographic and health	Gender	Male	360 (32%)
		Female	782 (68%)
	Age	<30 years	188 (16%)
		30–40 years	326 (29%)
		40–49 years	298 (26%)
		50–59 years	243 (21%)
		>60 years	87 (8%)
	Long-term limiting illness	No	1024 (90%)
		Yes	114 (10%)
	Difficulty walking quarter mile on the level	No	1123 (99%)
		Yes	17 (1%)
	Child in household aged under 5	No	970 (85%)
		Yes	165 (15%)
	Child in household aged 5–15	No	906 (80%)
		Yes	228 (20%)
SEP	Education	Degree or other	889 (78%)
		A-level	142 (12%)
		GCSE/none	107 (9%)
	Tenure	Owner occupied	848 (75%)
		Private rented	243 (21%)
		Social rented/other	47 (4%)
	Small-area income deprivation (fifths)^a^	1 (most affluent)	212 (19%)
		2	235 (21%)
		3	229 (20%)
		4	241 (21%)
		5 (least affluent)	224 (20%)
Distance	Commute distance	<3 km	122 (11%)
		3–4.9 km	322 (28%)
		5–9.9 km	216 (19%)
		10–20 km	179 (16%)
		20–30 km	188 (16%)
		>30 km	114 (10%)
Access to cars and parking	Driving licence valid in UK	No	110 (10%)
		Yes	1032 (90%)
	Household cars per adult	None	168 (15%)
		Less than one	526 (46%)
		One or more	443 (39%)
	Workplace parking	No parking	365 (32%)
		Paid parking	347 (31%)
		Free parking	417 (37%)

Numbers sometimes add to less than 1142 because of missing data; multiple imputation used to include all 1142 participants in regression analyses. For numbers stratified by car commuting type, see Electronic appendix available with the online version of the paper.

a Fifths defined with reference to the study population: in relation to England, fifth 1 corresponds to the 7% most affluent areas; fifth 2 to the 7–16% most affluent; fifth 3 to 16–33%; fifth 4 to 33–50%; and fifth 5 to the 50% least affluent areas.

**Table 2:** Predictors of regular car commuting among all study participants (*N* = 1142).

			% Regular car commuters	Risk ratios (95% CI)			
				Minimally-adjusted^a^	Multivariable 1^b^	Multivariable 2^b^	Multivariable 3^b^
Demographic and health	Gender	Male	26	1***	1***	1***	1***
		Female	45	1.80 (1.49, 2.18)	1.68 (1.40, 2.03)	1.50 (1.27, 1.77)	1.41 (1.21, 1.65)
	Age	<30 years	26	1***	1	1	1
		30–40 years	39	1.59 (1.21, 2.09)	1.17 (0.89, 1.53)	1.08 (0.87, 1.36)	1.06 (0.87, 1.30)
		40–49 years	40	1.67 (1.27, 2.20)	1.06 (0.80, 1.41)	0.99 (0.79, 1.24)	0.97 (0.80, 1.19)
		50–59 years	45	1.86 (1.41, 2.45)	1.13 (0.86, 1.50)	1.04 (0.83, 1.31)	0.98 (0.80, 1.19)
		>60 years	44	1.93 (1.38, 2.69)	1.09 (0.78, 1.53)	1.27 (0.96, 1.67)	1.18 (0.90, 1.54)
	Long-term limiting illness	No	37	1**	1*	1	1
		Yes	53	1.29 (1.06, 1.55)	1.23 (1.01, 1.49)	1.10 (0.95, 1.28)	1.15 (0.98, 1.36)
	Difficulty walking	No	38	1***	1*	1	1
		Yes	71	1.72 (1.26, 2.36)	1.43 (1.01, 2.04)	1.43 (0.99, 2.05)	1.19 (0.83, 1.70)
	Child aged under 5	No	39	1	1	1	1
		Yes	39	1.17 (0.94, 1.46)	1.11 (0.90, 1.38)	1.09 (0.90, 1.30)	1.03 (0.87, 1.22)
	Child aged 5–15	No	38	1	1	1	1
		Yes	43	1.09 (0.91, 1.31)	0.96 (0.80, 1.15)	1.04 (0.89, 1.21)	0.97 (0.84, 1.12)
SEP	Education	Degree or other	35	1***	1***	1	1
		A-level	51	1.36 (1.13, 1.65)	1.27 (1.06, 1.53)	1.03 (0.88, 1.20)	1.02 (0.88, 1.19)
		GCSE/none	60	1.49 (1.23, 1.80)	1.44 (1.19, 1.74)	1.06 (0.91, 1.24)	1.12 (0.96, 1.31)
	Tenure	Owner occupied	45	1***	1***	1	1
		Private rented	18	0.42 (0.31, 0.57)	0.47 (0.35, 0.63)	0.78 (0.61, 1.00)	0.94 (0.75, 1.18)
		Social rented/other	32	0.72 (0.47, 1.08)	0.71 (0.48, 1.04)	0.83 (0.57, 1.23)	1.12 (0.78, 1.61)
	Area income deprivation (fifths)	1 (most affluent)	31	1***	1***	1	1
		2	46	1.51 (1.19, 1.92)	1.44 (1.13, 1.82)	1.10 (0.91, 1.32)	1.12 (0.94, 1.35)
		3	59	1.84 (1.47, 2.31)	1.70 (1.36, 2.11)	1.05 (0.88, 1.26)	1.04 (0.88, 1.23)
		4	27	0.96 (0.73, 1.27)	0.95 (0.73, 1.24)	0.93 (0.75, 1.16)	0.99 (0.80, 1.22)
		5 (least affluent)	29	0.94 (0.71, 1.25)	0.90 (0.68, 1.18)	0.90 (0.72, 1.13)	0.96 (0.78, 1.19)
Distance	Commute distance	<3 km	2	0.05 (0.01, 0.21)		0.06 (0.01, 0.23)	0.09 (0.02, 0.34)
		3–4.9 km	7	0.22 (0.14, 0.33)		0.23 (0.15, 0.35)	0.28 (0.18, 0.43)
		5–9.9 km	33	1***		1***	1***
		10–20 km	68	1.97 (1.59, 2.44)		1.86 (1.50, 2.30)	1.65 (1.35, 2.02)
		20–30 km	71	2.11 (1.71, 2.60)		2.03 (1.65, 2.51)	1.83 (1.50, 2.23)
		>30 km	81	2.34 (1.90, 2.88)		2.27 (1.84, 2.82)	1.91 (1.56, 2.34)
Access to cars and parking	Driving licence	No	8	1***			1*
		Yes	42	4.85 (2.60, 9.07)			1.81 (1.02, 3.18)
	Household cars per adult	None	1	0.04 (0.01, 0.17)			0.10 (0.03, 0.39)
		Less than one	29	1***			1***
		One or more	65	2.11 (1.82, 2.45)			1.42 (1.25, 1.61)
	Workplace parking	No parking	24	1***			1***
		Paid parking	45	1.79 (1.44, 2.21)			1.22 (1.04, 1.44)
		Free parking	48	1.95 (1.59, 2.38)			1.38 (1.17, 1.61)

*p < 0.05,**p < 0.01,***p < 0.001, using tests for heterogeneity.

a Adjusted for gender and age.

b Adjusted for all variables in column.

**Table 3:** Predictors of unimodal and occasional car commuting.

			Predictors of predominantly untimodal car commuting among regular car commuters (*N* = 444)			Predictors of occasional car commuting non-regular car commuters (*N* = 697)		
			% Unimodal	Minimally-adjusted^a^: risk ratios (95% CI)	Multivariable^b^: risk ratios (95% CI)	% Occasional	Minimally-adjusted^a^: risk ratios (95% CI)	Multivariable^b^: risk ratios (95% CI)
Demographic and health	Gender	Male	73	1	1	35	1	1**
		Female	65	0.89 (0.77, 1.02)	0.94 (0.84, 1.06)	40	1.21 (0.99, 1.48)	1.31 (1.09, 1.58)
	Age	<30 years	67	1	1	29	1**	1
		30–40 years	66	0.97 (0.77, 1.22)	0.88 (0.73, 1.07)	34	1.22 (0.88, 1.69)	0.94 (0.67, 1.31)
		40–49 years	72	1.05 (0.84, 1.32)	0.98 (0.81, 1.20)	42	1.52 (1.11, 2.08)	0.88 (0.62, 1.24)
		50–59 years	64	0.93 (0.73, 1.19)	0.88 (0.71, 1.09)	48	1.73 (1.27, 2.38)	0.96 (0.68, 1.36)
		>60 years	63	0.92 (0.67, 1.26)	0.96 (0.75, 1.22)	43	1.60 (1.05, 2.43)	1.09 (0.72, 1.66)
	Long-term limiting illness	No	67	1	1	38	1	1
		Yes	68	1.05 (0.87, 1.26)	1.08 (0.90, 1.31)	44	1.10 (0.80, 1.51)	1.15 (0.86, 1.55)
	Difficulty walking	No	67	1	1	38	1***	1
		Yes	67	1.02 (0.67, 1.54)	0.96 (0.68, 1.35)	80	2.04 (1.38, 3.02)	1.39 (0.85, 2.26)
	Child aged under 5	No	65	1*	1	38	1	1
		Yes	78	1.20 (1.03, 1.41)	1.12 (0.97, 1.29)	39	1.17 (0.87, 1.56)	0.91 (0.68, 1.21)
	Child aged 5–15	No	65	1	1	36	1*	1
		Yes	72	1.07 (0.91, 1.25)	0.95 (0.83, 1.09)	48	1.26 (1.01, 1.58)	1.06 (0.85, 1.31)
SEP	Education	Degree or other	68	1	1	36	1	1
		A-level	67	0.99 (0.82, 1.19)	1.00 (0.85, 1.17)	47	1.20 (0.91, 1.56)	1.02 (0.78, 1.32)
		GCSE/none	63	0.94 (0.76, 1.15)	1.05 (0.89, 1.25)	51	1.22 (0.88, 1.69)	1.05 (0.78, 1.41)
	Tenure	Owner occupied	66	1	1	46	1***	1
		Private rented	72	1.09 (0.88, 1.36)	1.11 (0.91, 1.34)	24	0.53 (0.39, 0.72)	0.77 (0.58, 1.03)
		Social rented/other	73	1.14 (0.83, 1.56)	1.21 (0.86, 1.69)	16	0.33 (0.15, 0.73)	0.58 (0.30, 1.09)
	Area income deprivation (fifths)	1 (most affluent)	60	1	1	39	1	1
		2	70	1.22 (0.96, 1.56)	1.13 (0.92, 1.40)	46	1.17 (0.89, 1.54)	1.08 (0.83, 1.39)
		3	70	1.23 (0.97, 1.56)	1.11 (0.90, 1.36)	44	1.09 (0.81, 1.47)	0.89 (0.67, 1.18)
		4	64	1.12 (0.85, 1.47)	1.01 (0.80, 1.26)	35	0.93 (0.70, 1.24)	0.97 (0.75, 1.26)
		5 (least affluent)	68	1.20 (0.92, 1.58)	1.02 (0.81, 1.29)	32	0.80 (0.59, 1.08)	0.83 (0.62, 1.11)
Distance	Commute distance	<3 km	100	[combined†]	[combined†]	27	0.58 (0.41, 0.81)	0.77 (0.56, 1.06)
		3–4.9 km	87	1.36 (1.09, 1.70)	1.17 (0.92, 1.50)	29	0.61 (0.47, 0.78)	0.70 (0.56, 0.88)
		5–9.9 km	65	1*	1	48	1***	1***
		10–20 km	64	1.00 (0.80, 1.24)	0.94 (0.77, 1.14)	60	1.22 (0.93, 1.60)	1.15 (0.89, 1.49)
		20–30 km	66	1.01 (0.82, 1.25)	0.98 (0.81, 1.18)	59	1.21 (0.91, 1.60)	1.25 (0.94, 1.67)
		>30 km	68	1.06 (0.85, 1.32)	1.02 (0.83, 1.26)	59	1.19 (0.81, 1.77)	0.99 (0.69, 1.42)
Access to cars and parking	Driving licence	No	56	1	1	26	1*	1
		Yes	67	1.18 (0.65, 2.17)	0.89 (0.45, 1.73)	41	1.47 (1.04, 2.08)	0.85 (0.63, 1.16)
	Household cars per adult	None	100	[combined^c^]	[combined^c^]	8	0.19 (0.11, 0.32)	0.20 (0.12, 0.34)
		Less than one	67	1	1	42	1***	1***
		One or more	67	0.99 (0.86, 1.13)	1.02 (0.91, 1.14)	61	1.44 (1.21, 1.71)	1.30 (1.08, 1.55)
	Workplace parking	No parking	19	1***	1***	29	1***	1***
		Paid parking	64	3.36 (2.15, 5.26)	3.33 (2.14, 5.16)	41	1.41 (1.09, 1.81)	1.29 (1.02, 1.63)
		Free parking	90	4.76 (3.09, 7.33)	4.75 (3.11, 7.25)	49	1.70 (1.36, 2.14)	1.67 (1.35, 2.07)

*p < 0.05,**p < 0.01,***p < 0.001, using tests for heterogeneity.

a Adjusted for gender and age.

b Adjusted for all variables in column.

c Combined with group below due to small cell size (*N* = 2).
