# Supplementary material for: Healthy travel and the socio-economic structure of car commuting in Cambridge, UK: A mixed-methods analysis
Source: Soc Sci Med. 2012 Jun;74(12):1929–38. doi: 10.1016/j.socscimed.2012.01.042 (PMC3611603; doi:10.1016/j.socscimed.2012.01.042)
Supplement: Supplementary file 1 [file mmc1.doc]

Table 1: Demographic, health, socio-economic and work-related characteristics of study participants (N=1142 individuals), by car commute type

|  |  |  | **Total** | **By car commute type (N)** | | | |
| --- | --- | --- | --- | --- | --- | --- | --- |
|  |  |  | **sample (N)** | **Regular, unimodal** | **Regular, multimodal** | **Non-regular, occasional** | **Non-regular, never** |
| Demo- | Gender | Male | 360 | 69 | 25 | 94 | 172 |
| graphic |  | Female | 782 | 228 | 122 | 174 | 258 |
| and | Age | <30 years | 188 | 33 | 16 | 40 | 99 |
| health |  | 30-40 years | 326 | 84 | 43 | 68 | 131 |
|  |  | 40-49 years | 298 | 86 | 34 | 75 | 103 |
|  |  | 50-59 years | 243 | 70 | 40 | 64 | 69 |
|  |  | >60 years | 87 | 24 | 14 | 21 | 28 |
|  | Long-term | No | 1024 | 255 | 128 | 242 | 399 |
|  | limiting illness | Yes | 114 | 41 | 19 | 24 | 30 |
|  | Difficulty | No | 1123 | 289 | 142 | 264 | 428 |
|  | walking | Yes | 17 | 8 | 4 | 4 | 1 |
|  | Child in house- | No | 970 | 244 | 133 | 225 | 368 |
|  | hold aged under 5 | Yes | 165 | 51 | 14 | 39 | 61 |
|  | Child in house- | No | 906 | 225 | 120 | 200 | 361 |
|  | hold aged 5 to 15 | Yes | 228 | 70 | 27 | 63 | 68 |
| Socio- | Education | Degree or other | 889 | 208 | 99 | 212 | 370 |
| econ |  | A-level | 142 | 48 | 24 | 33 | 37 |
| omic |  | GCSE/none | 107 | 40 | 24 | 22 | 21 |
|  | Tenure | Owner occupied | 848 | 253 | 130 | 215 | 250 |
|  |  | Private rented | 243 | 31 | 12 | 47 | 153 |
|  |  | Social rented/other | 47 | 11 | 4 | 5 | 27 |
|  | Small-area | 1 (most affluent) | 212 | 37 | 28 | 58 | 89 |
|  | income | 2 | 235 | 76 | 33 | 58 | 68 |
|  | deprivation | 3 | 229 | 95 | 40 | 41 | 53 |
|  | (fifths)† | 4 | 241 | 44 | 25 | 61 | 111 |
|  |  | 5 (least affluent) | 224 | 45 | 21 | 50 | 108 |
| Dist- | Commute | <3km | 122 | 2 | 0 | 32 | 88 |
| ance | distance | 3-4.9km | 322 | 20 | 3 | 86 | 213 |
|  |  | 5-9.9km | 216 | 46 | 25 | 70 | 75 |
|  |  | 10-20km | 179 | 78 | 44 | 34 | 23 |
|  |  | 20-30km | 188 | 88 | 46 | 32 | 22 |
|  |  | >30km | 114 | 63 | 29 | 13 | 9 |
| Access | Driving licence | No | 110 | 5 | 4 | 26 | 75 |
| to cars | valid in UK | Yes | 1032 | 292 | 143 | 242 | 355 |
| and | Household | None | 168 | 2 | 0 | 13 | 153 |
| parking | cars per | Less than one | 526 | 103 | 51 | 157 | 215 |
|  | adult | One or more | 443 | 191 | 96 | 95 | 61 |
|  | Workplace | No parking | 365 | 17 | 72 | 81 | 195 |
|  | parking | Paid parking | 347 | 100 | 56 | 78 | 113 |
|  |  | Free parking | 417 | 180 | 19 | 107 | 111 |

Numbers in the first column sometimes add to less than 1142 because of missing data. † Fifths defined with reference to the study population: in relation to England, fifth 1 corresponds to the 7% most affluent areas; fifth 2 to the 7-16% most affluent; fifth 3 to 16-33%; fifth 4 to 33-50%; and fifth 5 to the 50% least affluent areas.

Table 2: Car commute type in relation to past-week commuting trips (N=1142 individuals)

|  | **Percentage of trips to and from work in the past week (%)** | | | | | | | | | |
| --- | --- | --- | --- | --- | --- | --- | --- | --- | --- | --- |
|  | **Car** | **Car+ bike** | **Car+ walk** | **Car+ PT** | **Car+PT+ bike/ walk** | **Bike** | **Walk** | **PT** | **PT+ bike/walk** | **Motorbike or other** |
| **All regular (N=444)** | 62 | 9 | 14 | 5 | 3 | 3 | <1 | 1 | 2 | <1 |
| - **Unimodal (N=297)** | 91 | <1 | <1 | <1 | <1 | 4 | <1 | 2 | 1 | <1 |
| - **Multimodal (N=147)** | 5 | 26 | 42 | 14 | 9 | 2 | <1 | 1 | 2 | <1 |
| **All non-regular (N=668)** | 6 | <1 | <1 | <1 | <1 | 64 | 11 | 6 | 10 | 1 |
| - **Occasional (N=268)** | 15 | 1 | 1 | <1 | 2 | 56 | 6 | 7 | 10 | 2 |
| - **Never (N=430)** | 0 | 0 | 0 | 0 | 0 | 70 | 14 | 5 | 10 | 1 |
| **TOTAL**  **SAMPLE (N=1142)** | 28 | 4 | 6 | 2 | 2 | 41 | 7 | 4 | 7 | 1 |

PT=public transport.

Table 3: Comparison of study population with all Cambridge adult residents

|  |  | **Cambridge region** | **Study population** | **Census 2001 data†** |
| --- | --- | --- | --- | --- |
| Demographic | Female | Cambridge city | 64% | 50% |
|  |  | Surrounding areas | 72% | 50% |
|  | Aged under 30 | Cambridge city | 21% | 40% |
|  |  | Surrounding areas | 10% | 22% |
| Socio- | Degree or equivalent | Cambridge city | 86% | 41% |
| economic | education | Surrounding areas | 72% | 22% |
| position | Housing privately rented | Cambridge city | 36% | 18% |
|  |  | Surrounding areas | 11% | 7% |
|  | Income deprivation | Cambridge city | 15% / 29% | 21% / 35% |
|  | (least deprived 5%  / most deprived 50%) | Surrounding areas | 17% / 13% | 10% / 18% |

† Census (2001) Standard Area Statistics (England and Wales) http://www.neighbourhood.statistics.gov.uk/dissemination

Table 4: Commute mode to work in relation to work start time (N=4855 journeys)

|  | **Percentage of trips to work at different times (%)** | | | | | | | |
| --- | --- | --- | --- | --- | --- | --- | --- | --- |
|  | **6-7.30** | **7.30-8** | **8-8.30** | **8.30-9** | **9-9.30** | **9.30-10** | **10 to 12** | **Other** |
| **Pure car (N=1277)** | 7 | 13 | 20 | 29 | 20 | 7 | 3 | 2 |
| **Multimodal car (N=653)** | 2 | 9 | 29 | 23 | 23 | 10 | 3 | 1 |
| **PT or PT+ bike/ walk (N=513)** | 7 | 12 | 26 | 26 | 12 | 7 | 7 | 2 |
| **Bike (N=2028)** | 3 | 4 | 15 | 27 | 31 | 11 | 7 | 2 |
| **Walk (N=336)** | 3 | 6 | 12 | 25 | 28 | 13 | 8 | 4 |
| **Other (N=48)** | 6 | 8 | 21 | 27 | 17 | 15 | 6 | 0 |
| **TOTAL SAMPLE (N=4855)** | 4 | 8 | 19 | 27 | 25 | 10 | 5 | 2 |

PT=public transport.
